# Region-Specific Characteristics of Astrocytes and Microglia: A Possible Involvement in Aging and Diseases

**DOI:** 10.3390/cells11121902

**Published:** 2022-06-12

**Authors:** Jae Lee, Sung Wook Kim, Kyong-Tai Kim

**Affiliations:** Department of Life Sciences, Pohang University of Science and Technology (POSTECH), Pohang 37673, Korea; cake4855@postech.ac.kr (J.L.); kimsw@postech.ac.kr (S.W.K.)

**Keywords:** astrocytes, microglia, regional heterogeneity, aging, neurodegenerative disease

## Abstract

Although different regions of the brain are dedicated to specific functions, the intra- and inter-regional heterogeneity of astrocytes and microglia in these regions has not yet been fully understood. Recently, an advancement in various technologies, such as single-cell RNA sequencing, has allowed for the discovery of astrocytes and microglia with distinct molecular fingerprints and varying functions in the brain. In addition, the regional heterogeneity of astrocytes and microglia exhibits different functions in several situations, such as aging and neurodegenerative diseases. Therefore, investigating the region-specific astrocytes and microglia is important in understanding the overall function of the brain. In this review, we summarize up-to-date research on various intra- and inter-regional heterogeneities of astrocytes and microglia, and provide information on how they can be applied to aging and neurodegenerative diseases.

## 1. Introduction

Astrocytes and microglia are major types of glial cells that support the homeostasis of neurons through various functions [1,2]. Astrocytes, which are the largest subclass of glial cells, participate in the formation of the blood–brain barrier (BBB), regulate blood flow as part of the cerebral vascular system, and mediate synapse formation, as well as their transmission [3,4,5,6]. In the case of microglia, which are the dominant immune cells of the central nervous system (CNS), they are critical for brain homeostasis, inflammatory response, neurodevelopment, and neurogenesis [2,7,8,9]. Customarily, both astrocytes and microglia have been thought to be a homogeneous population with a similar function throughout the brain region. Interestingly, with the development of RNA sequencing techniques, the heterogeneity of astrocytes and microglia has received pinnacle attention [10,11]. Numerous past studies showed that both astrocytes and microglia display either morphological or functional heterogeneity [12,13,14]. For instance, while some astrocytes, such as protoplasmic astrocytes, have complex and fine processes, others, such as fibrous astrocytes, have relatively simple and coarse processes [15]. In the case of microglia, functionally different populations of activated microglia with a continuum of phenotypes have been found under disease conditions [16]. On the other hand, the regional differences of astrocytes and microglia have started to gain attention only very recently.

The regional heterogeneity of astrocytes and microglia in the brain refers to their heterogeneity within the same brain region (intraregional heterogeneity) or the heterogeneity among different regions (inter-regional heterogeneity) [17]. Recent studies have shown that regionally distinct subpopulations of astrocytes and microglia are associated with aging and various neurodegenerative diseases [18]. In fact, the hallmarks of aging and neurodegenerative diseases, such as region-specific neurodegeneration or cortical volume decrease, have been found to be associated with the regional heterogeneity of astrocytes and microglia. Likewise, studies on the regional heterogeneity of astrocytes and microglia are critical in understanding the pathology of diseases and the brain as a whole. Although the functional heterogeneity of other glial cells, such as oligodendrocytes and ependymal cell, has been studied [19,20], only little is known about their regional distribution [21,22,23]. Thus, in this review, we summarize and focus on recent studies about the regional heterogeneity of astrocytes and microglial functions in the cortex, hippocampus, cerebellum, and other brain regions. Additionally, we provide insights on how the regional heterogeneity of astrocytes and microglia could be implicated in aging and neurodegenerative diseases.

## 2. Intra- and Inter-Regional Heterogeneity of Cortex-Specific Astrocytes and Microglia

The cortex is the largest part of the brain that is associated with higher-level processes, such as memory, motivation, and consciousness [24]. It also contains different functional regions dedicated to specific functions of our body, such as the somatosensory cortex and motor cortex that governs sensation and voluntary movement [25]. This functional and regional heterogeneity within the cortex led researchers to investigate the regional heterogeneity of astrocytes and microglia.

### 2.1. Cortex-Specific Astrocytes and Their Function

**Figure 1:**
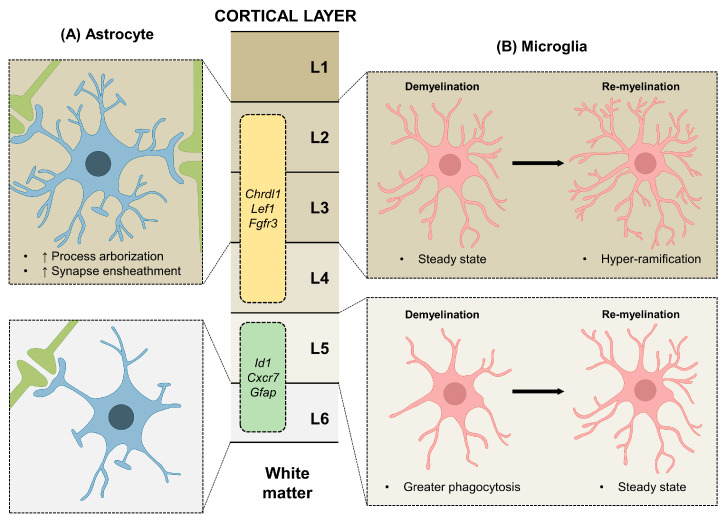
Intraregional heterogeneity of astrocytes and microglia in cortical layer. (**A**) Cortical layer 2/3 (L2/3) astrocytes (blue) have distinct characteristics when compared to L6 astrocytes. L2/3 astrocytes have greater process arborization and synapse ensheathment than the L6 astrocytes, leading to greater synaptic regulation. The molecular features of upper-layer astrocytes (ULAs, orange box) and deep-layer astrocytes (DLAs, green box) are shown. (**B**) Microglia (pink) in different cortical layers also show diverse responses to demyelination and remyelination. When demyelination is induced with cuprizone, a copper chelator, the microglia in L5 show increased phagocytosis, while L2/3 microglia remain at steady state. On the other hand, the L2/3 microglia display hyper-ramification under remyelination, while L5 microglia remain at steady state. *Chrdl1*: chordin-like 1; *Lef1*: lymphoid enhancer binding factor 1; *Fgfr3*: fibroblast growth factor receptor 3; *Id1*: inhibitor of differentiation-1; *Cxcr7*: chemokine receptor 7; *Gfap*: glial fibrillary acidic protein.

The cerebral cortex consists of six neuronal layers, each with different functions and connections of neural input and output. Numerous previous studies have reported that different neurons are present at each cortical layers [26,27,28,29], but in the case of astrocytes, only a small number of studies has demonstrated their heterogeneity in the cortical layers [30,31,32]. A recent study reported that astrocytes present in the mouse somatosensory cortex have a layer-specific morphology and molecular functions [30]. They showed that the morphology of the astrocytes in cortical layer 2/3 (L2/3) has a greater process arborization than that of the astrocytes in L6 [30]. Additionally, the cell orientation from the pia mater was different between L2/3 astrocytes and L6 astrocytes [30]. Not only that, astrocytes in L2/3 regulate more synapse ensheathment than astrocytes in L6 (Figure 1A) [30]. Furthermore, astrocytes from six cortical layers were largely divided into two regions, upper-layer astrocytes (ULAs, L2/3, and L4) and deep-layer astrocytes (DLAs, L5, and L6), and their gene expressions were analyzed through RNA sequencing [30]. Although most of the previously reported markers of astrocytes were expressed similarly in both regions, some of the genes from two regions showed significant differences [30]. For example, while the expression of fibroblast growth factor receptor 3 (*Fgfr3*), which is a negative regulator of the glial fibrillary acidic protein (*Gfap*) [33], chordin-like 1 (*Chrdl1*), and lymphoid enhancer binding factor 1 (*Lef1*) were elevated in ULAs, the level of *Gfap*, inhibitor of differentiation-1 (*Id1*), and chemokine receptor 7 (*Cxcr7*) were upregulated in DLAs [30]. Consistent with these results, a recent study also showed a functional heterogeneity of astrocytes found in different cortical layers [34]. Through a gene ontology (GO) analysis, the astrocytes found in the middle cortical layers (L2/3 and L4) were found to have functions related to the “steroid metabolic process” and “lipid metabolic process”, while the astrocytes in L5 and L6 were found to participate in the “cellular response to hormone stimulus” [34]. Altogether, the morphology, gene expression, and functions of astrocytes vary depending on their resident cortical layers.

Another recent study revealed that the cortical astrocytes form a distinct layer system that is different from the six neuronal layers [31]. The gray matter astrocytes from L2 to L6 were able to be classified into three layers: superficial, mid, and deep astrocyte layers [31]. Astrocytes from L1 and deep L6 were excluded because they showed characteristics of white matter astrocytes [31,35]. Each layer of gray matter astrocytes was characterized by the elevated expression of *Chrdl1*, SUMO-conjugating enzyme 1 (*Sce1*), and interleukin 33 (*Il-33*), respectively [31]. The astrocytes in the superficial layer that highly expressed *Chrdl1*, which increases the level of synaptic GluA2 AMPA (α-amino-3-hydroxy-5-methyl-4 isoxazolepropionic acid) receptors, control the synapse maturation and plasticity [36]. In accordance with this study, a recent report found astrocytes with similar molecular fingerprints in cortical layers [34]. Similar to astrocytes in the superficial layer, the *Chrdl1*^+^ astrocytes were found in middle cortical layers (L2/3 and L4) [34]. These astrocytes also showed a high expression of the solute carrier family 7 member 10 (*Slc7a10*) and glutamate ionotropic receptor AMPA-type subunit 2 (*Gria2*), which are important genes in glutamatergic neurotransmission [34]. On the other hand, *Il-33*^+^ astrocytes were found in L5 and L6, and were enriched with the gamma-aminobutyric acid type A receptor subunit gamma 1 (*Gabrg1*), an important gene for GABAergic neurotransmission. These molecular features seen in layer-specific astrocytes show a correlation to the types of neurons that comprise each cortical layer. Just like the middle cortical layer astrocytes that participate in glutamatergic neurotransmission, the dominant types of neurons in L2/3 and L4 are the excitatory stellate cells and pyramidal cells [37,38]. On the other hand, L5 and L6 contain more somatostatin^+^ interneurons that mediate GABAergic inhibitory signaling, which shows a correlation to astrocytes in L5 and L6 that are enriched with *Gabrg1* [39]. Furthermore, the formation of the astrocyte layer is affected by the neuronal cortical layer [30,31]. When the formation of the neuronal cortical layer was disturbed in special AT-rich sequence-binding protein 2 (*Satb2*), *Reeler*, and disabled-1 (*Dab1*) mutant mice [40,41,42], the layer-specific heterogeneity of astrocyte was also changed [30,31]. This means that the formation of the neuronal cortical layer during the brain development is critical in the formation of different morphologies and molecular functions of the cortex-specific astrocytes [30,31].

Cortex-specific astrocytes also have different characteristics depending on the functional areas of the cortex, as it did in the neuronal cortical layer. In a recent paper, the gene expression of astrocytes was different among the somatosensory cortex (SC), visual cortex (VC), and motor cortex (MC) of an adult mouse [43]. In the case of MC and VC astrocytes, the number of upregulated genes was higher than the other cortical astrocytes, while SC astrocytes showed the least region-specific gene expression [43]. The expression of the genes related to synaptic regulation also appeared differently among the functional regions of the cortex [43]. While VC astrocytes highly expressed genes such as *Gfap* and complement cascade components, MC astrocytes were enriched with genes related to synapse elimination or synaptic transmission, such as multiple EGF-like domains 10 (*Megf10*) and inwardly rectifying potassium channel (*Kir4.1*) [43].

### 2.2. Cortex-Specific Microglia and Their Function

Similar to astrocytes, microglia also show regional heterogeneity within and among the cortical regions. Although there have been some controversies on their heterogeneity [44], recent developments in the genetic analysis through single-cell RNA sequencing revealed the existence of cortex-specific microglia, both during development and in the adult human brain [10]. A recent study observed the regional heterogeneity of microglia in a developing human brain [45]. The single-cell RNA sequencing of microglia from 19 human embryo yolk sacs and head tissues revealed that there were 19 different microglial clusters [45]. Out of 19 clusters, the cortex-specific C7 cluster was considered “neuronal gene-enriched microglia”, as they showed an elevated expression of stathmin-2 (*STMN2*), SRY-box transcription factor 11 (*SOX11*), and tubulin beta 2A class IIa (*TUBB2A*) [45]. These microtubule-related genes in microglia are important for the ramification of the microglial processes, which are necessary for microglia–neuron crosstalk during neurogenesis [46,47]. The ramified microglia, which are the homeostatic microglia, are known to remove apoptotic neurons [48], or produce growth factors such as insulin-like growth factor 1 (IGF-1) and the brain-derived neurotrophic factor (BDNF), which could promote neurogenesis [49]. Li et al. (2022) further showed that the cortex-specific microglia were enriched with neuronal differentiation 2 (*NEUROD2*) and neuronal differentiation 6 (*NEUROD6*), which are important factors in brain development and neurogenesis, respectively [50,51,52]. The enrichment of these neuronal genes implicates that these microglia would be necessary for cortex-specific development and neurogenesis.

Another recent study also revealed cortex-specific microglia in the adult human brain [53]. Lopes et al. (2022) isolated microglia from four different regions of postmortem brains that were donated by 115 donors. The transcriptome analysis of these isolated microglia revealed that the microglia from two cortical regions, the medial frontal gyrus (MFG) and superior temporal gyrus (STG), were significantly different from the microglia in the subventricular zone (SVZ) and thalamus [53]. When the microglial populations were divided into clusters, the two cortical microglia belonged to cluster 1, which was enriched with genes related to homeostatic microglia [53]. The microglia in the MFG and STG showed a strong expression of the purinergic receptor P2Y_12_ (*P2RY12*), which is an important gene for the extension of microglial processes [54], and *CD36* and mannose receptor C-type 1 (*MRC1*), which are genes important for microglial phagocytosis [55,56]. The elevated expression of such genes implicated that the microglial phagocytic function is enhanced in cortex-specific microglia. The phagocytic function of microglia is an important factor for microglia–neuron crosstalk during synaptic engulfment or pruning [57], which actively happens in the cortex of children and juveniles, throughout to adults [58].

In the case of mice, since the cerebral cortex makes up 77% of the rodent brain mass [59], the regional heterogeneity of microglia within the cortex of mice seems inevitable. One study showed that various subtypes of microglia reside within different cortical layers of the mouse brain [60]. When Roufagalas et al. (2021) induced demyelination in the cortical region of a mouse brain using cuprizone, a copper chelator that induces the selective apoptosis of oligodendrocytes followed by the activation of microglia [61], microglia from different cortical layers showed distinguishable responses. The subset of microglia from L5 responded earliest to demyelination by demonstrating increased phagocytic activity [60]. However, the microglia from L2/3 remained steady during demyelination, but became hyper-ramified during remyelination. These facts imply their differential functions in tissue recovery (Figure 1B) [62]. In addition, apart from the cortical layer, the deeper part of the cortex has its own specific microglia population that carries out different mitochondrial and oxidative functions [63]. Altogether, these studies implicate that distinguishable subpopulations of cortex-specific microglia exist among different cortical layers.

## 3. Intra- and Inter-Regional Heterogeneity of Hippocampus-Specific Astrocytes and Microglia

The hippocampus, which is located in the temporal lobe of the brain, is part of the limbic system that mainly regulates learning and memory [64]. Although the size of the hippocampus is relatively small when compared to the cortex, its structure shows high complexity consisting of different regions, such as CA1~3, the dentate gyrus, and subgranular zone (SGZ) [65]. Each region of the hippocampus is dedicated to a specific function, such as memory consolidation in CA1, contextual fear memory in the dentate gyrus, and adult neurogenesis in the SGZ [66,67,68]. Recently, distinct subpopulations of astrocytes and microglia were found in a specific location of the hippocampus, participating in different functions.

### 3.1. Hippocampus-Specific Astrocytes and Their Functions

Not only does the hippocampus contain a large number of synaptic connections between numerous neurons and single astrocytes [69], it also shows a synaptic plasticity that is actively regulated by both microglia and astrocytes [70,71]. Therefore, it is widely reported that hippocampus-specific astrocytes have functions related to synaptic plasticity [72]. Huang et al. (2020) showed that nuclear factor I-A (NFIA), a transcription factor, is required to maintain astrocyte function in the hippocampus, but not in the cortex or olfactory bulb [73]. NFIA is a switch that changes neurogenic competency to glial competency, where NFIA-induced astrocytes further regulate synaptogenesis and neuroprotection [74]. NFIA is also found in astrocytes from multiple brain regions [69,75]. However, in a mouse with astrocyte-specific NFIA deficiency, astrocytes at the CA1 region of the hippocampus displayed a drastically reduced morphological complexity, decreased number of major branches, shortened total process length, and diminished Ca^2+^ activity, while astrocytes in the cortex, olfactory bulb, and brainstem showed only mild changes [73]. The reason for this difference is that the expression of another NFI family member varies depending on the brain region [73]. For example, in the case of olfactory bulb-specific astrocytes, they react less sensitively to NFIA deficiency, because NFIB could compensate the deficiency by regulating NFIA signaling [73]. In the hippocampus, synaptic plasticity needs to occur constantly to regulate memory consolidation [76]. This may be why hippocampus-specific astrocytes react more sensitively to NFIA signaling, as the hippocampus requires fine adjustments of synaptic plasticity during memory consolidation.

The hippocampus is densely innervated by serotonergic fibers [77], and stimulations of serotonergic neurons have been found to enhance the rate of learning [78]. According to a recent study, serotonin (5-HT) released by serotonergic neurons activates “5-HT4R-Gα13–RhoA signaling” in astrocytes [79]. The signaling increases the RhoA-dependent filamentous actin assembly, which changes the morphology of the astrocytes [79]. Astrocytes regulate the synaptic function through the ensheathment of numerous neuronal synapses [80]. That is, when the morphology of astrocytes changes, the synaptic circuits associated with them are also affected. Therefore, the modification of astrocyte morphology due to “5-HT4R-Gα13–RhoA signaling” could adjust the excitatory synaptic circuits. The expression of 5-HT4R is greater in the hippocampus-specific astrocytes than in the astrocytes from other brain regions [43]. Since serotonergic systems have multiple effects on learning and memory [81], it can be assumed that 5-HT-sensitive astrocytes, which adjust the synaptic plasticity in response to 5-HT, are highly present in the hippocampus.

**Figure 2:**
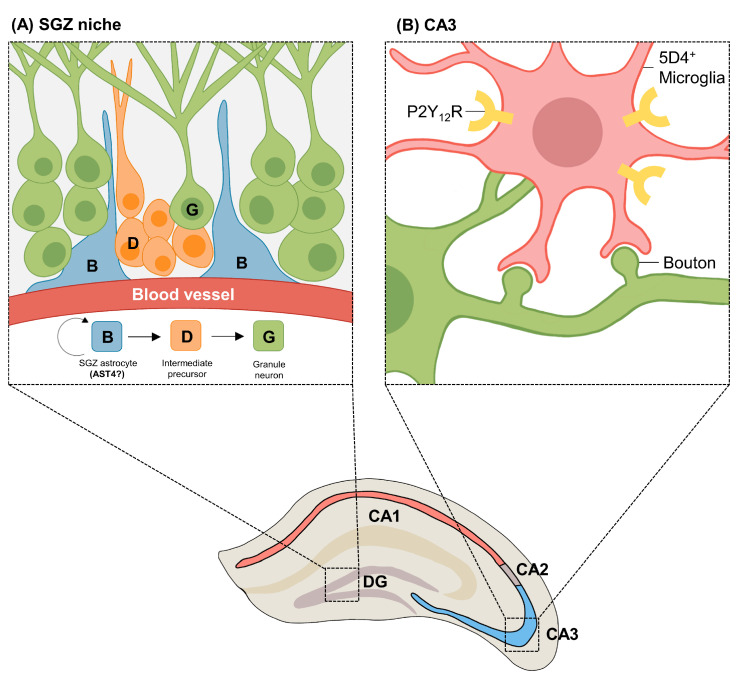
An example of hippocampus-specific astrocytes and microglia. (**A**) Adult neurogenesis in subgranular zone (SGZ) of hippocampal dentate gyrus (DG) is initiated when self-proliferate SGZ astrocyte (B, blue) divides into intermediate precursor cell (D, orange), which further differentiates into granule neurons (G, green). Batiuk et al. (2020) recently found that a specific subtype of astrocyte (AST4), which highly expressed genes associated with adult neurogenesis, was localized in SGZ. They hypothesized that AST4 may be the neural stem cell that participates in adult neurogenesis. (**B**) A specific subpopulation of microglia (pink) exists in CA3 region of hippocampus. 5D4 keratan sulfate epitope-positive (5D4^+^) microglia makes direct contacts with synaptic boutons of CA3 hippocampal neurons (green), thereby increasing the synaptic activity. CA3-specific 5D4^+^ microglia also highly express purinergic receptor P2Y_12_ (P2Y_12_R), which is known to regulate synaptic plasticity.

Another study observed astrocytes from the cortex and hippocampus using single-cell RNA sequencing and RNAscope to determine the regional heterogeneity of astrocytes [34]. The single-cell RNA sequencing data of astrocytes from the cortex and hippocampus of 56-day-old postnatal mice showed that there were five different astrocyte subtypes (AST) in the cortex and hippocampus [34]. Among them, the hippocampus-specific AST4 was localized largely in the SGZ located at dentate gyrus, and they highly expressed genes associated with cell fate specification (*Ascl1*) [82], and morphogenesis and differentiation (*Frzb*) [83], which are related to adult neurogenesis [68]. These data hypothesized that *Frzb*^+^*Ascl1*^+^*Slc1a3*^+^ AST4 represents a population of hippocampal neural stem cells or progenitor cells, which may be a subtype of astrocytes that participate in the SGZ-specific neurogenesis (Figure 2A) [84].

### 3.2. Hippocampus-Specific Microglia and Their Function

Since the hippocampus participates in specific functions of the brain, as previously discussed [66,67,68], the microglia that keep the homeostasis of the hippocampus should also have definite characteristics. Lawson et al. (1990) first suggested the existence of hippocampus-specific microglia, and since then, recent studies have supported its features [85,86]. As previously discussed, the hippocampus is a hub for adult neurogenesis that is crucial in maintaining healthy neurons in the brain [87]. One group recently demonstrated that a specific subpopulation of microglia in the hippocampus is crucial to adult neurogenesis during stressful conditions [88]. Under chronic mild stress, microglia with an elevated expression of arginase 1 (*Arg1*) are critical in maintaining the hippocampal neurogenesis and stress resistance [88]. The hippocampus-specific *Arg1*^+^ microglia are induced by interleukin 4 (IL-4) signaling, which in turn provokes BDNF-dependent neurogenesis [88]. The microglial BDNF is a critical factor of microglia–neuron crosstalk during different brain functions, as it binds to the neuronal tropomyosin-related kinase B (TrkB) receptor [89,90]. Thus, the existence of hippocampus-specific *Arg1*^+^ microglia would be necessary to maintain healthy neurogenesis in the hippocampus.

Likewise, a unique population of resting microglia was found in the CA3 region of the hippocampus. The CA3 region of the hippocampus has extensive internal synaptic connections when compared to other regions of the hippocampus, which demands the precise regulation of synaptic activity [91]. Interestingly, CA3-specific 5D4 keratan sulfate epitope-positive (5D4^+^) microglia were found to regulate synaptic activity in the CA3 region [92]. 5D4^+^ microglia were highly populated in the CA3 region of the hippocampus, making direct contact with synaptic boutons of CA3 hippocampal neurons [92]. This 5D4^+^ microglia may increase synaptic activity, as the previous study showed that direct contact between resting microglia and a synapse increases the synaptic activity [93]. Additionally, CA3-specific 5D4^+^ microglia showed an increased expression of interleukin-1β (IL-1β) and purinergic receptor P2Y_12_ (P2Y_12_R). Microglial IL-1β and P2Y_12_ are known to be important in the regulation of synaptic plasticity by either inducing synaptic loss or regulating neuronal excitability [94,95,96]. This implicates that CA3-specific 5D4^+^ microglia communicate with neurons to specifically regulate synaptic activity in the CA3 region of the hippocampus (Figure 2B).

A genetic analysis using a recent single-cell RNA sequencing technique also revealed a subpopulation of microglia with distinct profiles in the hippocampus [97]. Through analyzing the patterns of microglial genes using BioLayout Express^3D^ [98], three major clusters of genes were found in adult mouse microglia [97]. While different clusters of genes represented other regions of the brain, such as the cortex or cerebellum, the genes in cluster two were highly expressed in hippocampus-specific microglia [97]. Through a GO analysis, a great number of genes were found to be associated with the ‘generation of energy’ and ‘oxidative phosphorylation’ [97]. Specifically, peroxisome proliferator-activated receptor gamma (*Pparg*) and superoxide dismutase 1 (*Sod1*) were highly expressed in hippocampus-specific microglia [97]. PPAR-γ is an important factor that mediates the microglial activation [99]. A previous study showed that PPAR-γ activation in microglia could attenuate axonal injury [100]. Furthermore, microglial PPAR-γ may be important for bidirectional microglia–neuron crosstalk, as PPAR-γ can regulate glycoprotein CD200 expression on neurons [101], which, in turn, interacts with the CD200 receptor found only on microglia [102,103]. In the case of SOD1, SOD1 released by microglia is known to mediate neuroprotection under neurotoxic environments [104]. Together, these studies infer that the hippocampus-specific microglia found by Grabert et al. (2016) may communicate with neurons under situations that require neuroprotection.

## 4. Intra- and Inter-Regional Heterogeneity of Cerebellum-Specific Astrocytes and Microglia

The cerebellum, which is a major structure of the hindbrain, is located below the temporal lobe [105]. The cerebellum, which generates motor-related outputs and functions [106], is well-known for having distinct characteristics when compared to other brain regions [107]. For instance, the Purkinje cell (PC) is a unique type of neurons located in the cerebellar cortex that mediate output signaling from the cerebellum to other regions [108,109]. As such, a unique subpopulation of astrocytes and microglia would also be present in the cerebellum.

### 4.1. Cerebellum-Specific Astrocytes and Their Functions

**Figure 3:**
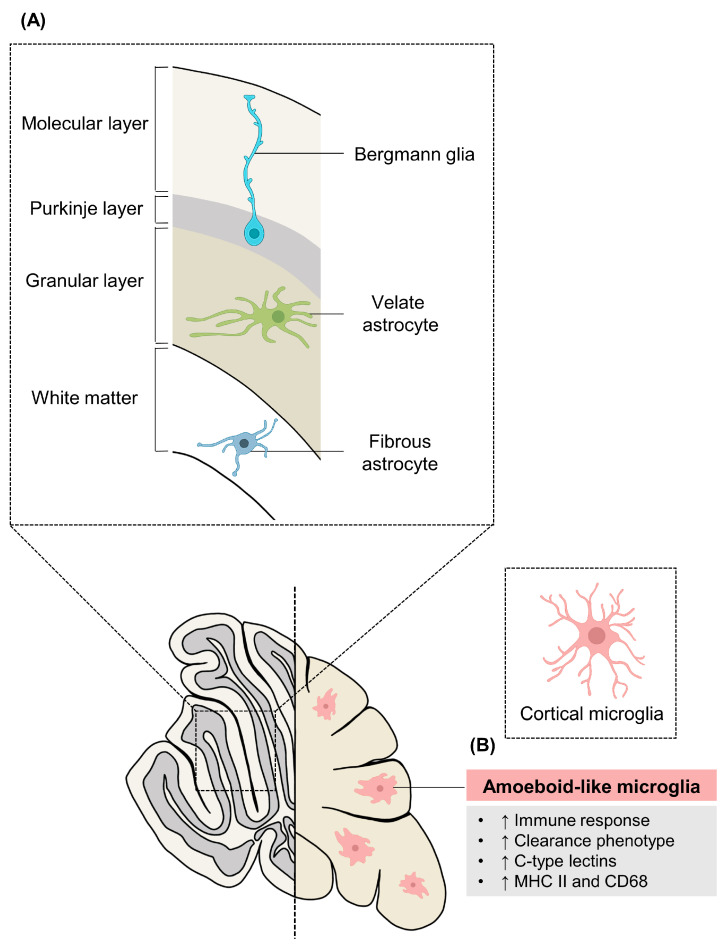
Intra- and inter-regional heterogeneity of cerebellum-specific astrocytes and microglia. (**A**) Three types of cerebellum-specific astrocytes are found in different layers of cerebellar cortex and white matter. Bergmann glia, which interact with Purkinje cells, are located in the molecular and Purkinje layer, while velate astrocytes are located in the granular layer. Fibrous astrocytes that regulate myelination and glutamate homeostasis are located in the white matter of cerebellum. (**B**) Cerebellum-specific microglia have distinct characteristics when compared to the cortical microglia. Morphology-wise, the cerebellum-specific microglia have smaller soma area, larger cytoplasm area, and lower ramification, mimicking amoeboid-like morphology. Functional-wise, the cerebellum-specific microglia show greater immune response and debris-clearance. MHC II, major histocompatibility complex II.

Cerebellum-specific astrocytes have unique characteristics unlike astrocytes from other brain regions. Nearly all synapses present in the cerebellum are ensheathed through astrocytic processes [110], while in the cortex and hippocampus, only approximately 50% or less synapses are ensheathed by astrocytes [111]. There is also intraregional heterogeneity in cerebellum-specific astrocytes, which could be largely divided into three types. Bergmann glia (BG) and velate astrocytes (VAs) exist in the cerebellar cortex, while fibrous astrocytes reside in the cerebellar white matter [112]. They have different morphologies, expressions of marker genes, and molecular functions [113]. For example, the cell body of BG, which is the most representative type of cerebellum-specific astrocytes, is located in the Purkinje layer, while its processes that ensheath PC dendrites and synapses are located in the molecular layer [114]. BG also adjust the migration of granule cells in early development [115], while interacting with PCs to regulate their maturation and the homeostasis of extracellular glutamate and other neurotransmitters in PC synapses during maturity as an adult [116,117]. Unlike BG, protoplasmic VAs with mossy fiber are located in the granular layer [117,118]. Although the exact function of VAs is yet to be known, it is assumed that they would be involved in regulating the function of granule cells, because they form a sheath with granule cells [119]. On the other hand, white matter astrocytes show a very low *Kir4.1* expression, adjust myelination, and engage in glutamate homeostasis (Figure 3A) [114,118]. Interestingly, the intraregional heterogeneity of astrocytes in the cerebellum is generated by an extrinsic cue, a sonic hedgehog (Shh) signaling expressed by mature neurons [114]. When the patched 2 receptor (Ptch2) of BG is heavily exposed to Shh produced by the mature PC, it maintains a high level of the AMPA receptor GluA1, GluA4, glial high-affinity glutamate transporter (GLAST), and a low level of water channel aquaporin 4 (AQP4) [114]. On the other hand, in the case of Vas, the expression of Ptch2 is low, and, thus, they are less affected by Shh, thereby maintaining the gene expression opposite to that of BG [114]. Furthermore, it was confirmed that activating Shh signaling on VAs changed its gene expression to be more like that of BG [114]. Therefore, the gene expression of certain astrocytes present in the cerebellum is not due to an intrinsic factor, but is caused by a neuronal cue [120].

### 4.2. Cerebellum-Specific Microglia and Their Functions

Different studies have also revealed that distinct populations of microglia might exist in the cerebellum. When Lawson et al. (1990) first found the heterogeneity of microglia, a significantly lower expression of F4/80 and morphological difference was observed in the cerebellum-specific microglia. In accordance with this finding, a recent study also showed morphological differences between cerebellum-specific microglia and microglia from the striatum, hippocampus, or cortex [121]. The cerebellum-specific microglia showed a smaller soma area, larger cytoplasm area, and lower ramification complexity, showing amoeboid-like morphology [121]. When the microglial transcriptome was analyzed with single-cell RNA sequencing, microglia in the cerebellum also showed very distinct profiles [97]. Cerebellum-specific microglia showed a high expression of C-type lectins, which are a group of proteins that recognize carbohydrates in a calcium-dependent manner [122], and a major histocompatibility complex class II (MHC II), a protein involved in antigen processing [97,123]. Accompanying the fact that these proteins participate in immune-related function [124,125,126], the GO analysis also demonstrated that genes with an increased cerebellum-specific microglia were related to the ‘immune response’ and ‘defense response’ [97]. Last, but not least, cerebellum-specific microglia displayed a disparate clearance phenotype when compared to microglia from other regions [127]. Cerebellum-specific microglia showed an increased expression of CD68 when compared to microglia in the striatum, which indicates an enhanced phagocytic activity in cerebellum-specific microglia (Figure 3B) [127,128,129].

## 5. Intra- and Inter-Regional Heterogeneity of Astrocytes and Microglia in Other Brain Regions

Apart from the cortex, hippocampus, and cerebellum, there are many other regions of the brain with specific functions. These other regions of the brain also have distinct characteristics and functions, which would require a unique population of astrocytes and microglia. Few recent studies focused on these subpopulations of astrocytes in the hypothalamus and thalamus, and microglia in the SVZ.

### 5.1. Hypothalamus-Specific Astrocytes and Their Functions

The hypothalamus senses the glucose level through hypothalamic glucose-sensing neurons [130] and further controls insulin sensitivity, glucose tolerance, and glucose production and uptake [131]. Hypothalamus-specific astrocytes are also involved in the glucose metabolism. A recent study revealed that hypothalamic astrocytes control the sensation and uptake of glucose across the BBB [132]. A hypothalamus-specific knockout of astrocytic insulin receptors (IRs) or postnatal ablation of IRs in GLAST-expressing cells altered astrocytes morphology and their several functions, such as circuit connectivity [132].

Another study showed that hypothalamus-specific astrocytes also expressed higher levels of synapse-modifying genes [43]. For example, secreted protein acidic and rich in cysteine (*Sparc*), which antagonizes synaptogenic functions of Hevin [133], was expressed higher in hypothalamus-specific astrocytes than that of the cortex [43]. Not only that, hypothalamus-specific astrocytes had 748 upregulated genes when compared to cortical astrocytes [43]. As a result of analyzing the overall function of upregulated genes through the GO analysis, it was found that hypothalamus-specific astrocytes highly expressed genes related to the “lipid and lipoprotein metabolism” [43].

### 5.2. Thalamus-Specific Astrocytes and Their Functions

Some studies have revealed that there are two subpopulations of astrocytes in the thalamus, that either express or lack the AMPA receptor (AMPAR) [134]. The function of thalamus-specific astrocyte changes depending on the existence of AMPAR [134]. The AMPAR-bearing astrocytes could lower the Kir current more than that of AMPAR-deficient astrocytes [134]. Interestingly, apart from thalamus-specific astrocytes, hippocampus-specific astrocytes show no AMPAR currents at all [135], while neocortex-specific astrocytes exhibit small AMPAR currents [136].

### 5.3. Subventricular Zone-Specific Microglia and Their Functions

Adult neurogenesis has been extensively studied over the years since its first discovery, which was more than 50 years ago [137,138,139]. Along with the hippocampus, the SVZ is a well-known niche for adult neurogenesis [140,141,142]. Due to its specific function in the brain, recent studies showed that a particular subpopulation of microglia might be involved in SVZ-specific neurogenesis [53,143,144]. One study demonstrated that microglia with a discrete morphology, such as having an amoeboid-like form and shorter branches, mediate SVZ neurogenesis [143]. They revealed that microglia had a lower expression of purinergic receptors, and showed decreased responses to ATP [143]. The depletion of such microglia in the SVZ impeded the survival and migration of neuroblasts, and, thus, the neurogenesis [143]. Additionally, the latest genetic analysis of microglial genes from a human postmortem brain showed that transcriptomes of SVZ-specific microglia were different from those in the cortical regions [53]. When the microglial population was divided into clusters, the microglia from the SVZ belonged to cluster four, which were enriched with genes related to hormonal signaling and interferon responses [53]. Specifically, interleukin-10 (*IL-10*) and clusterin (*CLU*), which are important factors in adult neurogenesis [145,146], were highly upregulated in SVZ microglia [53]. As such, the regional heterogeneity of microglia exists in the SVZ, accompanying the adult neurogenesis.

## 6. Could Regional Heterogeneity of Astrocytes and Microglia Be Implied in Aging?

Compared to a young brain, a normally aging brain shows various hallmarks, such as increased inflammation, the dysregulation of energy metabolism, and impaired lysosome function [147]. Additionally, the overall cortical volume is known to be decreased in normally aging brains. However, the degree of the hallmarks is shown differently among brain regions [147,148,149,150]. Since astrocytes and microglia are important mediators of aging hallmarks, their regional heterogeneity may affect the regional specificity of aging hallmarks.

### 6.1. Implication of Regional Heterogeneity of Astrocytes in Aging

One study observed region-specific changes of astrocytes in an aging brain by comparing astrocyte transcriptomes from mouse brains of 4 months (adult) and 2 years (aging) of age [43]. Astrocyte mRNAs were isolated using the RiboTag technique [151], and were then analyzed through single-cell RNA sequencing [43]. The following four regions of the brain were observed: the VC, MC, cerebellum, and hypothalamus. The overall trend in the aging brain displayed an increase in the immune pathway, consistent to previously found hallmarks of aging brains [43,147]. For example, the complement cascade, which is part of the immune system, was upregulated in astrocytes from all four regions of the brain [43]. This shows that astrocytes are also involved in the general increase in the aging-mediated immune pathway [43]. There were also region-specific changes in astrocytes in relation to the hallmarks of the aging brain [43]. Most of the genes related to cholesterol synthesis were mainly decreased in astrocytes from an aged hypothalamus [43]. Cholesterol is a structural component of the cellular membrane and plays an important role in maintaining the neuronal functions, such as synaptic vesicle formation, neurotransmission, and synapse formation [152]. Interestingly, locally produced cholesterol in the brain is very important, as the cholesterol cannot cross the BBB [153,154]. The cholesterol is synthesized by a master transcription factor, sterol regulation element-binding protein 2 (SREBP2) [155]. Generally, SREBP2 is highly expressed in astrocytes [155]. A large amount of cholesterol produced through SREBP2 forms a complex with apolipoprotein E (APOE) lipoprotein and moves to neurons to perform various functions [153]. However, in the astrocytes of an aging hypothalamus, the expressions of several target genes of SREBP2, such as 3-hydroxy-3-methylglutaryl-CoA reduction (*Hmgcr*), were significantly lowered [43]. Since hypothalamic neurons are important in regulating the energy homeostasis and stress response, the depletion of cholesterol in these hypothalamic neurons increases the risk of neurodegenerative diseases [156,157]. Therefore, promoting cholesterol synthesis in hypothalamus-specific astrocytes in the aging brain may prevent the degeneration of hypothalamic neurons, thereby lowering the risk of neurodegenerative diseases. Furthermore the genes related to synapse modification showed varying patterns of up- or downregulation by each brain region [43]. For example, transforming growth factor beta 2 (*Tgfb2*) was increased in the cerebellum of the aging brain, but was decreased in MC [43]. Altogether, region-specific hallmarks seen in aging brains are likely to be affected by regional the heterogeneity of astrocytes.

Boisvert et al. (2018) also confirmed that changes in astrocytic genes induced by aging were similar to the changes seen in reactive astrocytes, depending on the region. For example, the serpin family A member 3 (*Serpina3n*), which is an anti-chymotrypsin that is highly expressed by inflammation and nerve injury [158], was found to be upregulated in reactive astrocytes [159]. Similarly, astrocytic *Serpina3n* was also observed to be upregulated about four-folds higher in the VC region of the aging brain. *Gfap*, which is used as a marker for the gliosis of reactive astrocytes that appear in neurodegenerative diseases [160], is also upregulated in the aging brain. According to another similar study, the expressions of reactive astrocytic genes were also different among brain regions [161]. Through aging, the hippocampus and striatum, which are the regions that are most vulnerable to neurodegeneration, actually showed a higher activation of genes related to astrocyte reactivity [161,162]. Therefore, since aging and various neurodegenerative diseases have a similar impact on astrocytes [163], it seems inevitable to study them together.

### 6.2. Implication of Regional Heterogeneity of Microglia in Aging

Microglial heterogeneity is known to be at its greatest during the development of the brain [10]. Over the course of time, microglial heterogeneity tends to drop and their characteristics tend to show more uniformity [10,97]. However, a few studies demonstrated that microglial heterogeneity might show different responses to aging across the brain regions. When the microglial heterogeneity of the cortex, hippocampus, and cerebellum was compared at different ages (4 months, 12 months, and 22 months), the overall heterogeneity significantly dropped in the cortex and hippocampus [97]. The hippocampus-specific microglia lost their specificity and showed cortex-specific microglia-like characteristics by 22 months of age [97]. However, the cerebellum-specific microglia maintained their distinct characteristics even at 22 months of age [97], which implicates that the effect of aging is regionally different. The genetic analysis of microglial transcriptome also revealed a similar phenomenon, where most regions of the brain showed a similar transcriptomic change by aging, except for the SVZ [53]. While the expression of *MRC1* was downregulated in microglia from the cortex and thalamus, it was specifically upregulated in the SVZ microglial transcriptome [53]. This change in microglia seems to be correlated to the general hallmarks of aging. Previous reports showed that *MRC1* is a critical factor in regulating the microglial phagocytosis [56]. Interestingly, phagocytosis dysfunction is one of the hallmarks seen in microglia of the aging brain [164]. Thus, a decrease in the expression of *MRC1* across the brain region correlates to the decrease in the phagocytic function of microglia across the brain region. However, the increase in *MRC1* expression in SVZ-specific microglia may be associated with neurogenesis that actively happens in the SVZ [140,141,142]. In the aged brain, the level of interferon gamma (IFN-γ) is increased in the SVZ, and further activates microglia [165]. IFN-γ-activated microglia show an increase in the expression of *MRC1* [166], and further participate in neurogenesis [167]. Altogether, these studies implicate that although aging might have a uniform effect on microglial heterogeneity, some regions might respond differently, maintaining their regional heterogeneity.

## 7. Could Regional Heterogeneity of Astrocytes and Microglia Be Applied in Neurodegenerative Diseases?

In various neurodegenerative diseases, different symptoms, such as memory loss, anxiety, and mood disorders, are caused by neuronal death [168]. Neuronal death in the brain occurs in nonuniform patterns among the brain regions, causing previously mentioned symptoms of neurodegenerative diseases [169]. This nonuniformity in neurodegeneration could be explained by the regional heterogeneity of astrocytes and microglia, as they have a critical role in neurodegeneration [170,171]. Here, we described how the regional heterogeneity of astrocytes and microglia affect the regional neurodegeneration often seen in Alzheimer’s disease and Parkinson’s disease and other neurodegenerative diseases, such as amyotrophic lateral sclerosis and leukoencephalopathy.

### 7.1. Regional Heterogeneity of Astrocytes and Microglia in Alzheimer’s Disease

**Figure 4:**
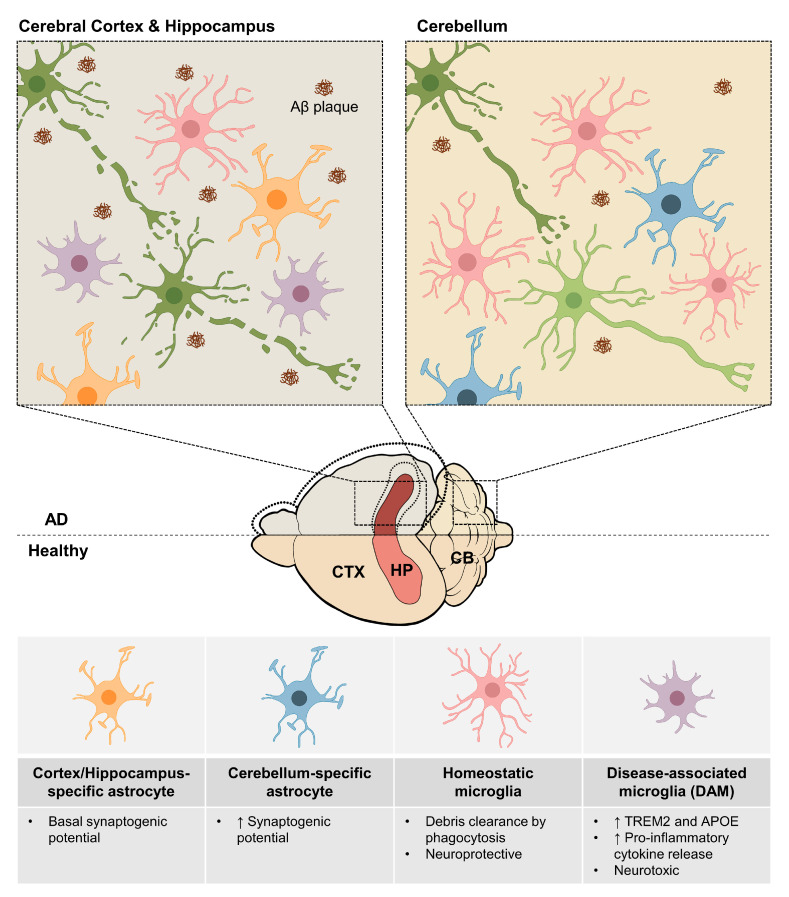
Regional heterogeneity of astrocytes and microglia during Alzheimer’s disease. During Alzheimer’s disease (AD), the volume of cerebral cortex and hippocampus tends to decrease due to neurodegeneration, while the volume of cerebellum shows minor changes (shown through dotted line). This discrepancy in the degree of neurodegeneration between cortex/hippocampus and cerebellum comes from the differences in the population of astrocytes and microglia in three regions. While the cortex/hippocampus-specific astrocytes (orange) show basal synaptogenic potential, the cerebellum-specific astrocytes (blue) show greater synaptogenic potential, which might prevent synaptic degeneration by amyloid β plaque (Aβ plaque, brown). With microglia, the number of homeostatic microglia (pink) with neuroprotective effect decreases in cortex and hippocampus, while the number does not change in the cerebellum. In addition, the disease-associated microglia (DAM, purple) with neurotoxic effect are only found in the cortex and hippocampus and not in the cerebellum. Thus, the degeneration of neuron happens at a greater degree in cortex and hippocampus when compared to cerebellum. CTX: cerebral cortex; HP: hippocampus; CB: cerebellum; ↑: increased.

Alzheimer’s disease (AD) is a neurodegenerative disease where neuronal death caused by amyloid β (Aβ) plaques and tau-containing neurofibrillary tangles (NFTs) leads to cognitive dysfunction [172]. Similar to other neurodegenerative diseases, the magnitude of degeneration in AD varies among different brain regions [173]. For example, the entorhinal cortex and hippocampus are most affected during AD, while the cerebellum shows relatively less defects [174]. One of the causes of this regional difference is the regional heterogeneity of astrocytes. One recent study demonstrated that the synaptogenic potential of astrocytes varies from region to region, so the effects of the disease are also different [112,175]. The quantification of specific pre- and postsynaptic proteins (synaptophysin/PSD-95) in neurons cultured with astrocyte-conditioned media from the cerebral cortex, hippocampus, midbrain, and cerebellum showed that cerebellum-specific astrocytes exhibited a higher synaptogenic potential than astrocytes from the other three brain regions [175]. Therefore, it can be assumed that the influence of Aβ plaques that cause synaptic degeneration would occur relatively less in the cerebellum than in other brain regions (Figure 4) [176]. The regional difference in the degree of neurodegeneration may also be affected by the accumulation of Aβ plaques. Aβ plaques are known to cause the chronic activation of microglia [177]. Chronically activated microglia secrete proinflammatory cytokines, such as interleukin -1α (IL-1α), tumor necrosis factor-α (TNFα), and complement component 1q (C1q), which further activate nearby astrocytes [178,179]. Thus, in AD, reactive astrocytes with different gene expressions, morphologies, and molecular functions are induced by the extrinsic cues from activated microglia. For example, complement component 3 (C3) is highly upregulated in reactive astrocytes and causes excessive immune responses [178]. Additionally, functions related to synapse formation and synapse phagocytosis seen in normal astrocytes are decreased in reactive astrocytes [178]. Furthermore, reactive astrocytes induce the degeneration of neurons and oligodendrocytes through the secretion of gamma-aminobutyric acid (GABA), TNF-α, nitric oxide (NO), lipocalin 2 (LCN2), and excessive fatty acid [180,181,182]. Since a previous study showed that the accumulation of Aβ plaques in the cerebellum happens significantly less than that in the cortex and hippocampus [183], the induction of reactive astrocytes by microglia would be lower in the cerebellum, which correlates to relatively less neurodegeneration in the cerebellum [174]. In this sense, inhibiting the level of microglial cytokines that induce reactive astrocytes may prevent reactive astrocyte-mediated neurodegeneration in other brain areas. In fact, one recent study reported that the transition from normal astrocytes to reactive astrocytes can be repressed through the inhibition of microglial cytokines by increasing the activity of the Glucagon-like peptide-1 receptor (GLP-1R) [184]. Thus, using the GLP-1R agonist, like NLY-01 used in the previous study, could potentially ameliorate the reactive astrocyte-mediated neurodegeneration [184]. Altogether, depending on the regional heterogeneity of astrocytes, the pathological symptoms may vary from region to region in the brain.

Region-specific astrocytes may also be induced by the region-specific pathogenesis of AD. NFTs, formed by the accumulation of hyper-phosphorylated tau, are initiated in the entorhinal cortex and gradually spread to the hippocampus and other regions of the cortex [185]. This pathological spreading pattern is closely related to the cognitive decline that appears in AD [186]. The cause of the accumulation of tau involves various mechanisms, one of which is the autophagy–lysosomal pathway [187,188,189]. If the lysosomal pathway is defected by aging, the aberrant tau cannot be degraded, resulting in the accumulation of tau [188,190,191]. The accumulated tau in neurons spread trans-synaptically among neurons [192]. In the case of astrocytes, they uptake tau located in the synapse when synapse degradation occurs [193]. When the tau uptake occurs, the expression of the transcription factor EB (TFEB) increases in the astrocyte [194]. This implicates that astrocytes in the entorhinal cortex would show an increased expression of TFEB. In fact, one study revealed that astrocytes in the entorhinal cortex of AD patients showed an upregulated expression of TFEB [191]. TFEB is the master regulator of lysosomal biogenesis, which further promotes the uptake and clearance of extracellular tau [194]. Martini-Stoica et al. (2018) used AAV-GFAP-TFEB to increase the expression of TFEB astrocytes specifically in the PS19 tauopathy mouse model and observed the tau pathology. As a result, the engulfment of phospho-tau by astrocyte was increased, reducing tau pathology and gliosis in the mouse hippocampus [194]. NFTs are characterized by region-specific discovery rather than brain-wide accumulation during the early disease stages [185]. Therefore, if the TFEB expression of astrocytes present in the entorhinal cortex could be adjusted, the spread of tau onto the hippocampus and other regions of the cortex could be suppressed to some extent.

Previous research has revealed that characteristics of microglia, including their morphology, density, and activation status, are significantly changed during AD [195,196,197]. However, whether that change occurs differently among the brain regions still needs to be determined. Recently, a specific subpopulation of microglia has been observed in the brain of 5xFAD mice, a mouse model of AD [198]. This subpopulation of microglia, which are called disease-associated microglia (DAM), showed an increased expression of triggering receptor expressed on myeloid cells 2 (TREM2) and APOE [198]. Interestingly, a previous study also revealed that DAM were specifically found in the cortex and hippocampus and not in the cerebellum [198]. The regional heterogeneity of DAM is likely due to the region-specific environmental cues that induce DAM. The transition from homeostatic microglia to DAM during AD occurs in a two-step process by environmental cues [199]. Homeostatic microglia pass through intermediate stage one DAM before the transition into stage two DAM [200]. Although the transition from homeostatic microglia to stage one DAM is currently unknown, the transition from stage one DAM to stage two DAM is known to be caused by TREM2 signaling [199]. Under disease conditions, different ligands of TREM2, such as Aβ plaques, activate TREM2 signaling in stage one DAM [201]. The activated TREM2 signaling sustains the microglial activation of stage one DAM through prosurvival pathways, such as β-catenin and mTOR pathways, and further induces the transition to stage two DAM [202,203]. Since Aβ plaques show a region-specific deposition [204], the TREM2 signaling that induces DAM would also happen regionally. This regional heterogeneity of DAM may be the reason why the cortex and hippocampus show more a severe neurodegeneration than the cerebellum during AD. Although the function of DAM is still controversial [199], recent studies focused on the detrimental role of DAM during AD [205,206,207]. The increased expression of TREM2 and APOE in DAM is associated with neurodegenerative phenotypes, such as the release of proinflammatory cytokines [206,208]. Thus, the regional heterogeneity of neurodegenerative microglia could be associated with the regional heterogeneity of AD pathology, although further research needs to be conducted (Figure 4). Some scientists also see proinflammatory DAM as potential therapeutic targets of AD [207]. The proteins related to proinflammatory DAM were increased in the brains of AD patients and showed a positive correlation to the progression of AD [207]. Thus, when the induction of pro-inflammatory DAM was inhibited by the suppression of TREM2 signaling through the ShK-223 peptide, the clearance of Aβ plaques was increased in the AD mouse model [207]. Additionally, when the number of DAM was reduced by the treatment of colony-stimulating factor 1 receptor (CSF1R) inhibitor, amyloid-related pathology was ameliorated, further confirming the therapeutic relevance of proinflammatory DAM in AD [209].

### 7.2. Regional Heterogeneity of Astrocytes and Microglia in Parkinson’s Disease

**Figure 5:**
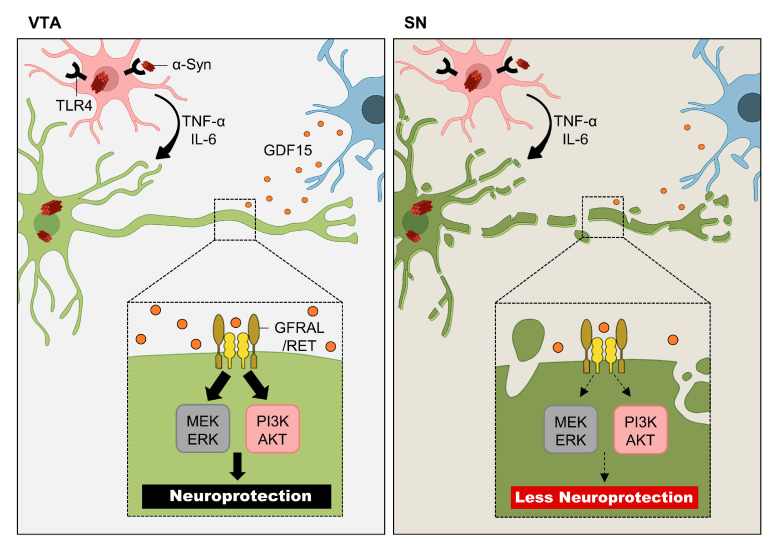
Implication of astrocytic and microglial regional heterogeneity in Parkinson’s disease. Substantia nigra (SN)-specific degeneration of dopaminergic neuron during Parkinson’s disease (PD) could be explained by the regional heterogeneity of astrocytes and microglia. The midbrain-specific microglia (pink) tend to show immune-alerted phenotype, highly expressing Toll-like receptor 4 (TLR4). Once the microglia uptakes α-synuclein through TLR4, it releases different pro-inflammatory cytokines, such as tumor necrosis factor-α (TNF-α) and interleukin-6 (IL-6) that are toxic to neuron. In ventral tegmental area (VTA), astrocytes (blue, left panel) release significant amount of growth differentiation factor 15 (GDF15), which binds to GFRAL/RET complex (GDNF-family receptor α-like/rearranged during transfection) and causes its activation. The activated GFRAL/RET complex then further activates extracellular signal-regulated kinase (ERK) and protein kinase B (AKT) signaling pathways that mediate neuroprotection. On the other hand, astrocytes in SN (blue, right panel) release significantly less (230-fold) GDF15. This results in less activation of ERK and AKT signaling and, thus, less neuroprotection.

Parkinson’s disease (PD) is a neurodegenerative disease that occurs when the dopaminergic (DA) neurons of the substantia nigra pars compacta (SN) selectively die by α-synuclein-rich Lewy bodies [210,211]. It is not fully understood why the defect occurs only in the SN, but it may be the result of the regional heterogeneity of astrocytes. Astrocytes in the ventral tegmental area (VTA), located right around the SN, express growth differentiation factor 15 (GDF15) about 230-fold higher than astrocytes in the SN [212]. These GDF15-expressing astrocytes exhibited the neuroprotection of both rat midbrain and iPSC-derived DA neurons through activating the extracellular signal-regulated kinase (ERK) and protein kinase B (AKT) pathway [212,213,214,215]. Therefore, it can be assumed that α-synuclein aggregation induces greater DA neuronal cell death in the SN than the VTA, as VTA-specific astrocytes show a greater GDF15-induced neuroprotection while SN-specific astrocytes show less neuroprotection (Figure 5).

With microglia, a specific subtype of microglial population was found in the nigrostriatal pathway, which could be relevant to the progression of PD [216]. Through a GO analysis, the midbrain-specific microglia from a healthy mouse brain showed an immune-alerted phenotype, such as an increased expression in inflammatory markers [216]. The transcriptome profiles of these microglia were rather similar to that of reactive microglia often seen during chronic inflammation [216,217]. This may be the reason why the neurons in the midbrain are more susceptible to degeneration, as the reactive microglia found in chronic inflammation tend to have a neurotoxic effect [218]. More importantly, the midbrain-specific microglia were enriched with Toll-like receptor 4 (TLR4) [216], which is a receptor known to uptake α-synuclein and further activate microglia [219]. Previously, the activation of microglia through the uptake of α-synuclein by TLR4 was found to be important in neurodegeneration during PD, as the activated microglia tend to release proinflammatory cytokines, such as TNF-α and interleukin-6 (IL-6) [220,221]. A lack of TLR4 in a mouse model of PD showed significantly less microglial activation, and, thus, the attenuation of neurodegeneration [220]. Having an elevated expression of TLR4 would indicate that midbrain-specific microglia are more sensitive to α-synuclein compared to microglia from other brain regions, which may be the reason behind the significant neurodegeneration in the nigrostriatal pathway during PD (Figure 5).

### 7.3. Regional Heterogeneity of Astrocytes and Microglia in Other Neurodegenerative Diseases

Amyotrophic lateral sclerosis (ALS) is a disease where the degeneration of upper and lower motor neurons in the motor cortex and spinal cord causes defects in motor control [222]. Recent studies showed that a transcriptomically distinct population of astrocytes with a neurotoxic phenotype was found in the corticospinal tract of somatic nervous systems in both an ALS mouse model and patients [223]. The astrocytes in the corticospinal tract showed an increased level of connexin 43 (Cx43) and high-mobility group box protein 1 (HMGB1) and a reduced level of glutamate transporter-1 (GLT-1), which are associated with neurotoxic effects [224,225,226]. Especially, an increase in the level of HMGB1 could activate the Toll-like receptor/receptor for advanced glycation end-products (TLR/RAGE) signaling pathways, which further promotes inflammation and motor neuron degeneration [227]. Furthermore, a decreased level of GLT-1 in astrocytes implies a disruption in the glutamate uptake, which may lead to neurodegeneration induced by glutamate excitotoxicity [228]. In this sense, increasing the level of GLT-1 in corticospinal astrocytes in ALS patients could be a potential therapeutic treatment. In fact, a previous report showed that the treatment of an ALS mice model with β-Lactam antibiotics increased the expression and activity of GLT-1, attenuated motor defects, and further increased the survival of these mice [229].

The intraregional heterogeneity of microglia was seen in the cortical layers of patients diagnosed with hereditary diffuse leukoencephalopathy with spheroids (HDLS) [230]. HDLS is a disease caused by mutations in the *CSF1R* gene, often described by white matter degeneration [231,232]. Interestingly, the number and activity of microglia in the frontal cortex of HDLS patients did not increase, unlike the phenotypes seen in microglia from other disease controls [230]. However, the microglia in L3 and L4 of the frontal cortex showed a uniform morphology and distribution, while the microglia in L5 and L6 and underlying white matter showed an uneven distribution, displaying an intraregional heterogeneity of microglia in human cortical layers [230]. The affected microglia in L5 and L6 and underlying white matter could be the reason behind the white matter degeneration during HDLS. Additionally, this heterogeneity could be due to the CSF1R signaling in microglia. CSF1R is a cell-surface receptor that mediates important signaling pathways associated with survival, maintenance, and the proliferation of microglia [233,234]. Previous reports showed that ligands of CSF1R, CSF-1, and interleukin-34 (IL-34), showed region-specific expression [235]. While the CSF-1 is largely expressed in the cerebellum, IL-34 is dominantly expressed in the cortex. As such, the ligands of CSF1R could be differentially expressed among the cortical layers, mediating different responses under HDLS conditions.

## 8. Conclusions

Astrocytes and microglia with distinct molecular signatures, morphology, and functions exist across different regions of the brain. In many cases, this regional heterogeneity of astrocytes and microglia is induced by environmental cues. Interestingly, particular subpopulations of astrocytes and microglia are associated with different region-specific hallmarks of aging. Some of them are also involved in the pathogenesis and regional defects of different neurodegenerative diseases. Despite the fact that the regional heterogeneity of astrocytes and microglia broadens the understanding of the brain, more precise research must be conducted to fully understand them. Many of the studies conducted involved a genetic analysis performed through single-cell RNA sequencing. Although the competency of the method has been checked through numerous past studies, it could be critical in the future to confirm the molecular features through in vivo studies. Additionally, a majority of the studies utilize different mice models of neurodegenerative diseases that only mimic the pathologies of actual diseases. Thus, the regional heterogeneity of astrocytes and microglia should be analyzed in actual human patients. Lastly, the difference in the regional heterogeneity of astrocytes and microglia between males and females must be analyzed in the future.

## Data Availability

Not applicable.
